# Noni enhances the anticancer activity of cyclophosphamide and suppresses myelotoxicity and hepatotoxicity in tumor-bearing mice

**DOI:** 10.1007/s00432-024-05734-1

**Published:** 2024-04-25

**Authors:** Mohammad Ali, S. N. Manjula, Ishfaq Mohiuddin, K. Mruthunjaya, Faiyaz Shakeel, Suhail Ahmad Mir, Shahid Ud Din Wani

**Affiliations:** 1 Department of Pharmacology, JSS College of Pharmacy, JSS Academy of Higher Education and Research, Mysuru, 570015 India; 2 Department of Pharmacology, Sri Adichunchanagiri College of Pharmacy, Sri Adichunchanagiri University, B.G Nagar, Bellur, Karnataka 571418 India; 3 Department of Pharmaceutical Sciences, School of Applied Science and Technology, University of Kashmir, Srinagar, 190006 India; 4 Department of Zoology, Annamalai University, Annamalainagar, 608 002 India; 5 Department of Pharmacognosy, JSS College of Pharmacy, JSS Academy of Higher Education and Research, Mysuru, 570015 India; 6 Department of Pharmaceutics, College of Pharmacy, King Saud University, 11451 Riyadh, Saudi Arabia

**Keywords:** Cancer, Toxicity, Antioxidant, Natural product, Divine noni

## Abstract

**Background and aim:**

*Morinda citrifolia* fruit juice (noni) is an herbal remedy documented to have antioxidant properties. It has been suggested that prevention of carcinogen-DNA adduct formation and the antioxidant activity of NJ may contribute to the cancer preventive effect. In the present study, the antitumor activity of noni was investigated in the presence of cyclophosphamide (CYL) in vitro and in vivo.

**Methods:**

In vitro breast cancer cells (MDA-MB-468) were used to measure the percentage of inhibition and the IC_50_. The in vivo antitumor activity of noni was studied by monitoring the mean survival time (MST), percentage increase in life span (%ILS), viable and non-viable cell count, tumor volume, body weight, and hematological and serum biochemical parameters in mice. Treatment with noni and CYL exhibited dose- and time-dependent cytotoxicity toward breast cancer cells.

**Results:**

Individual treatment of noni and CYL exhibited dose- and time-dependent cytotoxicity on breast cancer cell lines, while in combination therapy of noni and CYL, noni enhances cytotoxic effect of CYL at 48 h than that at 24 h. Similar result was found in in vivo studies, the results of which revealed that alone treatment of CYL and noni suppressed tumor growth. However, combination treatment with CYL and noni presented better tumor inhibition than that of alone treatment of CYL and noni. On the contrary, CYL alone drastically attenuated hematological parameters, i.e., RBC, WBC, and Hb compared to normal and control groups, and this change was reversed and normalized by noni when given as combination therapy with CYL. Moreover, the levels of serum biochemical markers, i.e., AST, ALP, and ALT, were significantly increased in the control and CYL-treated groups than those in the normal group. In the combination treatment of noni and CYL, the above biochemical marker levels significantly decreased compared to CYL alone-treated group.

**Conclusions:**

The present study suggested that CYL treatment can cause serious myelotoxicity and hepatic injury in cancer patients. In conclusion, the combined use of noni with CYL potentially enhances the antitumor activity of CYL and suppresses myelotoxicity and hepatotoxicity induced by CYL in tumor-bearing mice.

## Introduction

Cyclophosphamide (CYL) is a DNA alkylating agent commonly used in cancer treatment. Active CYL metabolites can suppress cancer cells (Ahlmann and Hempel 2016; El-Emam 2021; Natsuko et al. 2010). The effect of CYL on T cells is complex and dose dependent, and T cell lymphopenia and immunomodulatory effects can develop into immunosuppressive effects at higher doses (Huyan et al. 2011; Gladstone et al. 2002). Furthermore, CYL causes oxidative stress by generating free radicals (Roohi et al. 2017), which can lead to significant side effects, such as bone marrow suppression and hemorrhagic cystitis (Deng et al. 2018). Thus, supplementary therapies could increase the antitumor effect and decrease its toxic effects of CYL (Friery et al. 2000; Malaya et al. 2004). The edible noni plant has been used for more than 2000 years by Polynesian people as an herbal medicine for various kinds of ailments, i.e., arthritis, infection, diabetes, hypertension, asthma, and pain (Wang et al. 2002a). Scientifically, it is termed as *Morinda citrifolia* Linn. and is commercially known as noni. It was initially derived from the two Latin words “morus” attributed to mulberry and “indicus,” family Rubiaceae (Jain et al. 2012). It contains more than 160 chemical constituents, of which 120 are nutraceuticals; a large number of important phytochemical constituents have been recognized in the noni plant as well as in the fruit such as scopoletin, anthraquinones, octoanoic acid, vitamin C, terpenoids, b-sitosterol, polysaccharide, flavone glycosides, linoleic acid, carotene, alizarin, gallic acid, rosmarinic acid, amino acids, acubin, L-asperuloside, ursolic acid, quercetin, rutin, and proxeronine (Gajanan et al. 2017; Ali et al. 2021; Ayunda et al. 2020).

The anthraquinones, i.e., damnacanthal, nordamnacanthal, morindone, rubiadin, and rubiadin-1-methyl ether, anthraquinone glycoside present in noni has been documented as anticancer activity (Liu et al. 2000). Another literature reported that damnacanthal is a potent anthraquinone present in noni fruit and roots, and it can inhibit the growth of tumors by inhibiting the growth of activated genes (Hiramatsu et al. 1993) or by inducing a high rate of apoptosis in human colorectal cancer cell lines (Nualsanit et al. 2012). The components present in noni fruit, i.e., alizarin, have been reported to have antiangiogenic effects by obstructing blood circulation to tumors; limonene, one of the important compounds, averts mammary, lung, and liver cancers by activating the thymus gland to release additional T cells, which kill carcinoma cells; and ursolic acid, which averts the development of cancer-infected cells and induces cell death through increasing the immune system (Lv et al. 2011). The literature has reported that flavonoid compounds, triterpenoids and steroids, have manifold biological activities owing to their antioxidant properties. These phytochemical constituents reportedly inhibit the growth of cancer cells and protect against heart disease and different pathologies (DeFeudis et al. 2003; Takeoka and Dao 2003).

Several scientific studies have suggested that noni juice (NJ) prepared from noni fruit has several cancer-protective effects. Oncostatic accomplishments associated with cancer prevention include a decrease in TPA- or epidermal growth factor-induced cell transformation (Liu et al. 2001). It has been suggested that prevention of carcinogen-DNA adduct formation and the antioxidant activity of NJ may contribute to the cancer preventive effect (Wang et al. 2002b) and concentration-dependent free radical scavenging effects (Su et al. 2005; Pawlus et al. 2005). Likewise, they have additional anticancer properties, including antiangiogenic (Hornick et al. 2003) and cancer cell-selective cytotoxic properties (Arpornsuwan and Punjanon 2006; Hirazumi et al. 1994). Some authors have reported that NJ contains high levels of phenolic compounds and antioxidant capacity, thereby showing some level of cytotoxic activity in HeLa cell line (Janice et al. 2021). It has been reported that NJ increased mammary gland differentiation and reduced mammary tumor growth in mice (Clafshenkel et al. 2012). An earlier study presented that NJ/cisplatin by themselves and their combination were able to decrease lipid peroxidation and increase catalase activity in both the cervical cancer HeLa and SiHa cell lines. Hence, it can be concluded that NJ offers potential to be used as a chemoadjuvant and as an antioxidant, especially for the treatment of cervical cancer (Gupta and Singh 2013).

Noni juice (NJ) is most popular throughout the world because of its nutraceutical as well as therapeutic properties. Divine noni gold (DNG) is the preparation available in the market, which contains fresh NJ and extract of *Garcinia cambogia* fruit and liquorice. *Garcinia cambogia* is an herbal remedy belonging to the family Guttiferae (Clusiaceae), which has numerous pharmacological activities. Literature suggests that the fruit of *Garcinia cambogia* contains various phytoconstituents such as garcinol, isogarcinol, and xanthone compounds, i.e., oxyguttiferone M, oxyguttiferone K, oxyguttiferone K2, and oxyguttifer- one I which possess antioxidant activity (Mohammad Ali et al. 2021).

However, until now, no study has claimed that divine noni enhances anticancer activity in combination with CYL. Therefore, the present study was designed to investigate the adjunctive anticancer properties of divine noni in the presence of CYL in vitro and in vivo and to determine whether it could attenuate the myelotoxicity and hepatotoxicity induced by CYL treatment.

## Materials and methods

### Materials

Dulbecco’s Modified Eagle’s medium (DMEM-HiMedia Laboratories), fetal bovine serum (FBS-Gibco), phosphate-buffered saline (PBS-Thermo Fisher Scientific), dimethyl sulfoxide (DMSO-HiMedia Laboratories), sulforhodamine-B stain (SRB-Sigma Aldrich), human breast cancer cells (MDA-MB-468), propylene glycol, sodium chloride, methyl violet, trypan blue, and sodium sulfate (MERCK Limited, Mumbai, India) were used. All the other chemicals, including the reagents used, were of utmost analytical grade. The 1 g injection of marketed cyclophosphamide monohydrate was obtained from JSS Hospital, Mysuru, India.

### Noni products

In the present study, test samples such as fresh noni juice (NJ) and marketed divine noni gold (DNG) were used. Ripe noni fruits, as well as DNG, were obtained from Noni Biotech Pvt. Ltd. (Tamil Nadu, India). DNG is a commercial preparation that contains NJ, extracts of *Garcinia cambogia* fruit, and *Glycyrrhiza glabra* as a sweetening agent. DNG was packed in an 800-ml amber-colored plastic bottle. Fresh NJ was prepared from entirely cleaved noni fruit by hand press in the Pharmacology Laboratory of JSS College of Pharmacy, Mysuru, India, for use as the test sample. The noni products, i.e., NJ and DNG, used as test samples were kept frozen throughout the study.

### Qualitative phytochemical screening

The noni fruit extract was subjected to qualitative analysis to identify its constituents, including alkaloids, saponins, tannins, flavonoids, terpenoids, phlobatanins, proteins, and carbohydrates, following standard protocols (Mohammad Ali et al. 2021; Chew et al. 2011; Kumar et al. 2009; Harborne 1998).

### Identification and quantification of bioactive compounds using GC‒MS

The noni fruit extract was analyzed by a GC‒MS/MS-7000D Agilent (Agilent Technologies, Santa Clara, CA, USA) instrument equipped with an Agilent J&W GC‒MS column (HP-5Ms; 30 m × 250 mm × 0.25 μm). The column flow was held constant at 1 mL He/min, with an inlet temperature of 280 °C. The interface temperature was 250 °C, and the quadrupole temperature was 200 °C. Tenfold dilution of noni fruit extract injection volume of 1 μL was injected in split-less mode, with oven temperatures programed at 60 °C for 2 min, which was gradually raised with a gradient of 5 °C/min until 280 °C for 20 min, and then the gradient was set to initial, 20 °C/min for 3 min. By comparing their mass spectra to data from the National Institute of Standards and Technology (NIST) library, the compounds were identified.

### Anti-proliferative activity assessment by the SRB assay

To optimize the doses of NJ, DNG, and CYL for anticancer activity, the cytotoxicity of NJ and DNG toward breast cancer cells (MDA-MB-468) was evaluated by a sulforhodamine-B (SRB) assay in which breast cancer cells (10^3^ cells/well) were seeded in each well of a 96-well plate after they were instantly placed in a CO_2_ incubator (Thermo Fisher Scientific) for 24 h to adhere to the cells. Following adherence, the cells were treated with several concentrations of NJ, DNG, or CYL to determine the dose‒response effect of the individual therapies for 24 h and 48 h. Different concentrations of NJ and DNG were 0.3125, 0.625, 1.25, 2.5, 5, and 10 mg, and CYL at different concentrations, 2.18, 5.62, 11.25, 22.5, 45, and 90 µM, was used in the current SRB assay. Following treatment, the cells in the 96-well plate were gently washed 5 times with cold 10% (w/v) trichloroacetic acid solution, after which the 96-well plate was allowed to air dry at room temperature. After drying, the cells were stained with 0.4% SRB dye and incubated for 30 min. The plates were gently washed 4 times with 1% (v/v) CH_3_COOH (acetic acid) solution to eliminate SRB staining. After that, the plates were air-dried, and 10 mM aqueous TBS was added at a pH of 10.5. The absorbance was read at 540 nm by a microplate reader. The percentage of inhibition of cell growth by NJ, DNG, and CYL was calculated by the following formula, and the IC_50_ was calculated. The experiment was performed in triplicate.

$${\text{\% }}\;{\text{of}}\;{\text{inhibition }} = \frac{{{\text{Absorbance }}\;{\text{of}}\;{\text{control }} - {\text{ Absorbance}}\;{\text{of}}\;{\text{test}}}}{{{\text{Absorbance}}\;{\text{of}}\;{\text{control}}}}{ } \times 100$$

The combination of NJ or DNG with CYL at different concentrations, as described below, was tested for 48 h:

- CYL-11.25 µM + NJ-0.625 mg and CYL-11.25 µM + NJ-1.25 mg
- CYL-11.25 µM + DNG-0.625 mg and CYL-11.25 µM + DNG-1.25 mg

### Animals

Animal care, as well as management, was performed following the WHO guidelines, Geneva, Switzerland, and INSA (Indian National Science Academy, New Delhi, India), and was reported in accordance with the ARRIVE guidelines. The congenital Swiss albino mice were procured from the Central Animal House facility of JSS Medical College, Mysuru, India, and were used for the in vivo study. Seven to ten-week-old animals 26–30 g in weight were obtained from an inbred cluster that was well maintained under controlled humidity (50 ± 5%), temperature (23 ± 2 °C), and light (14 and 10 h of light and dark, respectively) conditions at the Central Animal Research Facility, JSS College of Pharmacy, Mysuru, India. All the mice were provided hygienic food and water ad libitum. Mice in the different groups were accommodated in polypropylene cages containing sterile paddy husks for bedding. The study was approved by the Institutional Animal Ethical Committee (IAEC No. 162/2016) of JSS College of Pharmacy, JSS University, Mysuru, India.

### Transplantation of tumors

EAC (Ehrlich ascites carcinoma) cells, procured from the Biochemistry Laboratory, JSS Medical College, Mysuru, India, were well preserved in the ascetic form by sequential passages in mice via weekly intraperitoneal transplantations of 2 × 10^6^ EAC cells. From the stock, a suspension of 0.1 ml of EAC cell suspension containing 1,000,000 EAC cells was injected intraperitoneally into the mice to induce the development of ascites tumors on day 0 in all the approved clusters, and each cluster contained 16 mice (Hiroshi et al. 1987). After 24 h of tumor inoculation, the mice were subjected to the following treatment schedule.

### *Treatment schedule (n* = *16)*

- **Group 1:** Normal group: Received vehicle.
- **Group 2:** Control: EAC cells were i.p. injected (1,000,000 cell/mouse; same for all groups).
- **Group 3:** Received CYL (40 mg/kg b.wt.) via i.p. injection for 4 days (Hiroshi et al. 1987).
- **Group 4:** Received NJ (360 mg/b.wt.) orally once a day for 2 weeks.
- **Group 5:** Received DNG (360 mg/b.wt.) orally once a day for 2 weeks.
- **Group 6:** Received CYL (40 mg/kg b.wt.) i.p. injection for 4 days + NJ (360 mg/b.wt.) orally once a day for 14 days.
- **Group 7:** Received CYL (40 mg/kg b.wt.) i.p. injection for 4 days + DNG (360 mg/b.wt.) orally once a day for 2 weeks. A volume of 0.35 ml of the equivalent to 360 mg was used in the experiment.

At the end of treatment, 10 mice from each group were used for the anticancer study, and another six mice from each group were used for myelotoxicity and hepatotoxicity studies. For the myelotoxicity and hepatotoxicity studies, blood was collected via heart puncture and through the carotid vein for the assessment of hematological and serum biochemical parameters.

### Antitumor activity measurements (*n* = 10)

The antitumor activity of the noni was determined in EAC tumor-bearing mice by estimating the following parameters:

#### Determination of the mean survival time (MST) and percentage increase in life span (%ILS)

After completing the treatment, the impact of NJ, as well as DNG, on tumor progression was checked by assessing the animal’s mortality every day for 60 days to evaluate the mean survival time, and the percentage increase in life span was calculated using the following formula:

$${\text{MST}} = \frac{{{\text{Day}}\;{\text{of}}\;{\text{first}}\;{\text{death}} + {\text{Day}}\;{\text{of}}\;{\text{last}}\;{\text{death}}}}{2}$$

$$\% {\text{ILS}} = \frac{{{\text{MST}}\;{\text{of}}\;{\text{the}}\;{\text{treated}}\;{\text{group}}}}{{{\text{MST}}\;{\text{of}}\;{\text{the}}\;{\text{control}}\;{\text{group}}}} - 1 \times 100$$

#### Viable and non-viable cell counts

A trypan blue assay was performed to evaluate the cells. The cells were stained with trypan blue dye (containing 0.4% trypan blue in normal saline). The non-viable cells are death cells which turn blue with trypan blue dye. The living cells are viable cells which do not take up the dye. The viable and non-viable cells were counted by the following formula (Asis et al. 2010):

$${\text{Cell}}\;{\text{count}} = { }\frac{{{\text{No}}.\;{\text{of}}\;{\text{cells }} \times {\text{ Dilution}}\;{\text{factor}}}}{{{\text{Area}} \times {\text{Thickness}}\;{\text{of}}\;{\text{liquid}}\;{\text{film}}}}$$

#### Determination of tumor volume

EAC fluid was collected from the peritoneal cavity via a disposable syringe and transferred to a graduated centrifuge tube to measure the tumor volume (Singh et al. 2013).

#### Body weight analysis

The body weight of the mice was measured at the beginning of the experiment (day 0) and successively on every 7th day throughout the duration of the treatment. The percentage decrease in body weight was calculated by the following formula (Mazumder et al. 1997):

$$\% {\text{Change}}\;{\text{in}}\;{\text{body}}\;{\text{weight}} = \frac{{{\text{Animal}}\;{\text{weight}}\;{\text{on}}\;{\text{respective}}\;{\text{day}}}}{{{\text{Animal}}\;{\text{weight}}\;{\text{on}}\;{\text{day}}\;0}} - 1 \times 100$$

### Toxicological studies (*n* = 6)

#### Hematological parameters

Hematological parameters such as RBC (D’Armour et al. 1965), WBC (Wintrobe et al. 1961), and Hb (Reitman and Frankel 1957) levels were estimated by standard procedures.

#### Biochemical estimation

Serum biochemical parameters, i.e., AST, ALT, and ALP, were estimated by the Reitman and Frankel method (Mingyue et al. 2021).

### Statistical analysis

Statistical analysis was completed by one-way analysis of variance (ANOVA) followed by Student’s *t* test. The values were expressed as the mean ± SEM. *p* < 0.05 was considered to indicate statistical significance.

## Results

### Phytochemical screening

Phytochemicals, which have been used as medicines for thousands of years, frequently play a crucial role in plant defense mechanisms against predators, stressors, and microorganisms. Thus, the first step in determining the types of potentially active molecules found in these plants and supporting their use as a traditional medicine is phytochemical screening. The ethanolic extract of *Morinda citrifolia* was utilized in the present study to screen for various phytochemicals. These included flavonoids, tannins, saponins, terpenoids, alkaloids, and steroids (Table 1).

**Table 1:** Different classes of phytochemicals present in the ethanolic extract of *Morinda citrifolia*

Phytoconstituents	Ethanolic extract of *Morinda citrifolia*
Flavonoids	++
Tannins	++
Saponins	+
Alkaloids	−
Steroids	++
Terpenoids	+

++: abundant, +: moderate, −: absent

### GC‒MS analysis of the ethanol extract of Morinda citrifolia

**Fig. 1:**
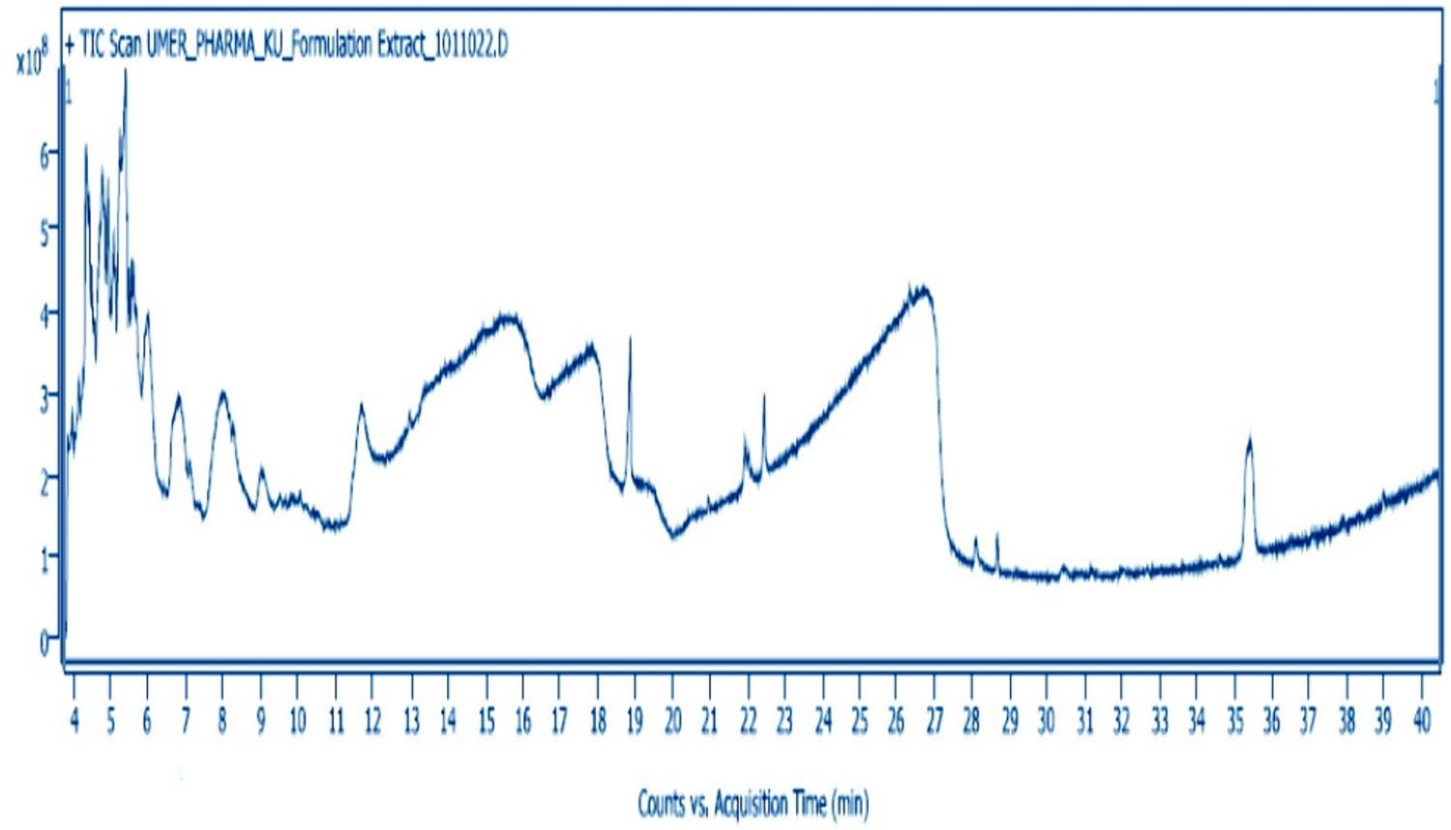
GC‒MS chromatograms of significant DNG compounds from *Morinda citrifolia*

A gas chromatograph‒mass spectrometer was used to isolate and identify 18 different compounds from the ethanolic extract of *Morinda citrifolia* by comparing their mass spectrum fragmentation patterns to those of well-known compounds listed in the NIST database (Fig. 1). The compounds identified in Table 2 are arranged by retention time, peak area (%), and molecular weight. The major compounds detected in the noni fruit extract were 1,3,2-oxazaborolane-4-carboxylic acid, 2-butyl-methyl ester, L- (22.03%), 4-methyl-1,2,3-thiadiazol-5-yl)methanol (19.56%), diethylborinic acid, a TMS derivative (10.69%), n-hexadecanoic acid (8.64%), benzoic acid, silver (1 +) salt (6.07%), and 3,3-dimethyl-5-phenyl-3H-pyrazole (5.26%). Pino et al. (2010) used headspace solid-phase microextraction to separate 96 compounds from noni fruit at two phases of ripening.

**Table 2:** GC‒MS analysis of the ethanolic extract of *Morinda citrifolia*

S. no.	Phytocompounds	Molecular formula	Molecular weight (g/mol)	Retention time (min)	CAS number	Peak area ± SD (%)
1	.beta, D-Mannofuranoside, methyl-2,3-O-(ethylboranediyl)-	C_9_ H_17_ B O_6_	232.04	3.845	1000149-59-6	1.17 ± 0.02
2	(4-Methyl-1,2,3-thiadiazol-5-yl)methanol	C_4_ H_6_ N_2_ O S	130.169	3.968	163008-86-4	1.93 ± 0.01
3	.alpha.-d-Xylopyranoside, 2,4-0-(ethylboranediyl)-1-0-methyl-	C_8_ H_15_ B O_5_	202.0	4.139	61553-47-7	0.53 ± 0.00
4	Diethylborinic acid, TMS derivative	C_7_ H_19_ B O Si	158.12	4.336	746330-37-8	10.69 ± 0.01
5	3,3-Dimethyl-5-phenyl- 3H-pyrazole	C_11_ H_12_ N_2_	172.23	4.410	5363-10-0	5.26 ± 0.06
6	Berteroin	C_7_ H_13_ N S_2_	175.3	4.491	4430-42-6	2.08 ± 0.01
7	Benzoic acid 1-methoxy-1H-tetrazol-5-ylmethyl ester	C_10_ H_10_ N_4_ O_3_	234.21	4.843	1000296-08-2	1.82 ± 0.00
8	Benzoic acid, silver(1+) salt	C_7_ H_5_ Ag O_2_	228.98	5.392	532-31-0	6.07 ± 0.00
9	1,3,2-Oxazaborolane-4-carboxylic acid, 2-butyl-,methyl ester, L-	C_8_ H_16_ BN O_3_	185.03	6.002	31970-40-8	22.03 ± 0.01
10	(4-Methyl-1,2,3-thiadiazol-5-yl)methanol	C_4_ H_6_ N_2_ O S	130.169	6.848	163008-86-4	19.56 ± 0.01
11	4,5,6,7-Tetrahydroxydecyl isothiocyanate	C_11_ H_21_ NO_4_ S	263.36	7.110	57103-43-2	0.17 ± 0.00
12	3-Hexene, 3- diethylboryl-4-trimethylsilyl	C_13_ H_29_ B Si	224.27	9.525	59766-51-7	0.19 ± 0.02
13	n-Hexadecanoic acid	C_16_ H_32_ O_2_	256.42	18.871	57-10-3	8.64 ± 0.00
14	.alpha.-D-Glucopyranoside, 1-0-methyl-2,3-0-diethylboryl-4,6-0-octylidene	C_23_ H_46_ B_2_ O_6_	440.2	20.951	1000158-77-2	0.31 ± 0.06
15	D-Mannitol	C_6_ H_14_ O_6_	182.17	25.921	69-65-8	0.04 ± 0.01
16	Polygalitol	C_6_ H_12_ O_5_	164.16	26.338	1000126-63-2	0.20 ± 0.03
17	Adamantane, 1-isothiocyanato-3-methyl-	C_12_ H_17_ N S	207.34	28.670	136860-48-5	0.79 ± 0.01
18	Tris(tert-butyldimethylsilyloxy)arsane	C_18_ H_45_ As O_3_ Si_3_	468.7	38.989	1000366-57-5	0.49 ± 0.00

### Cytotoxicity study by an SRB assay

#### Percentages of MDA-MB-468 cell inhibited by NJ, DNG, and CYL

**Fig. 2:**
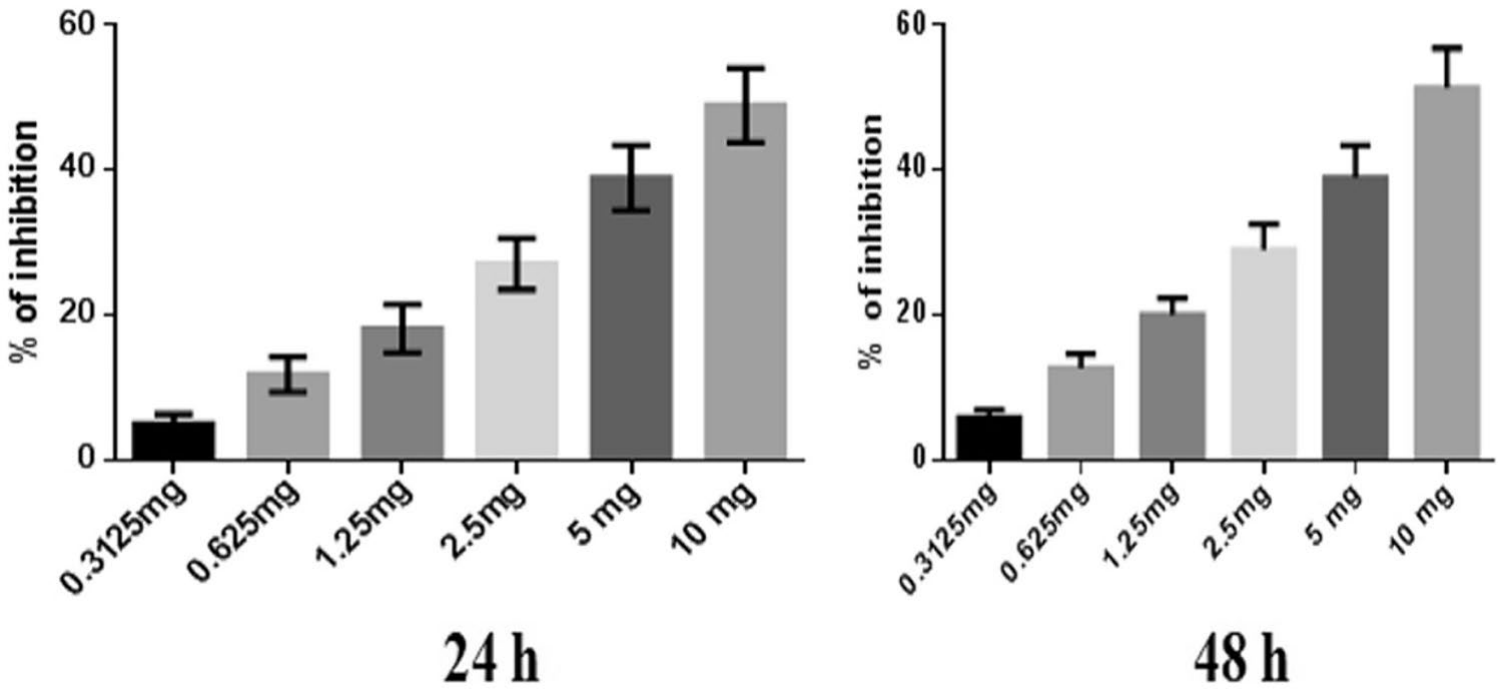
Anticancer efficacy of NJ on breast cancer cells at 24 h and 48 h. All values are displayed as the mean ± SEM of triplicate samples. The data were analyzed by one-way ANOVA followed by Tukey’s test

NJ, DNG, and CYL had dose- and time-dependent cytotoxic effects on cancer cells. The percentage of inhibition of NJ at a maximum concentration of 10 mg showed 48.93 ± 5.1% and 51.3 ± 5.5% inhibition at 24 h and 48 h, respectively. The IC_50_ of NJ was found to be 9.17 mg and 8.75 mg at 24 h and 48 h, respectively (Fig. 2).

**Fig. 3:**
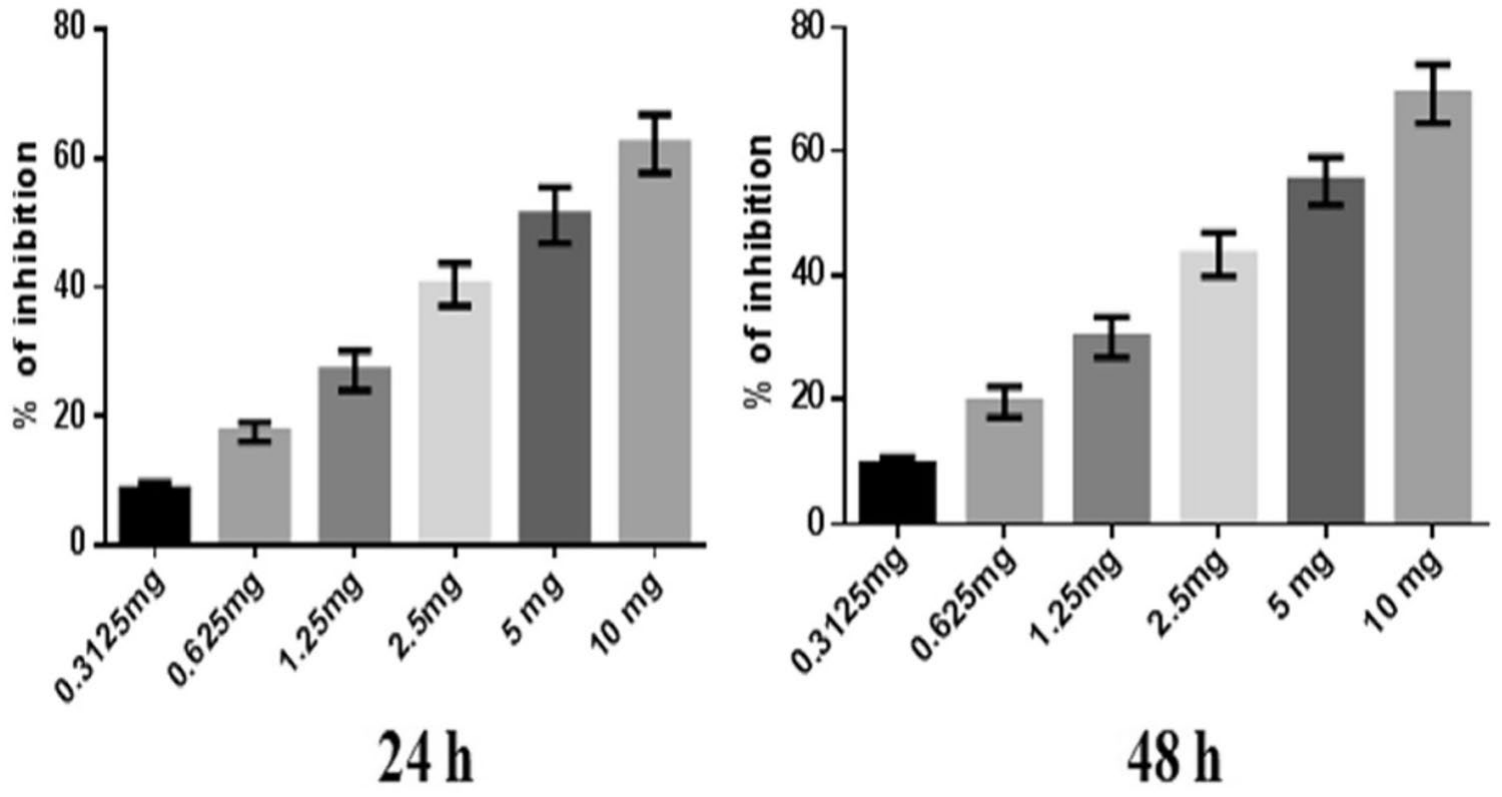
Anticancer efficacy of DNG on breast cancer cells at 24 h and 48 h. All values are displayed as the mean ± SEM of triplicate samples. The data were analyzed by one-way ANOVA followed by Tukey’s test

Similarly, the percentage of inhibition of DNG at a maximum concentration of 10 mg showed 62.29 ± 4.5% and 69.29 ± 4.7% at 24 h and 48 h, respectively. The IC_50_ of DNG was found to be 6.33 mg at 24 h and 5.44 mg at 48 h (Fig. 3).

**Fig. 4:**
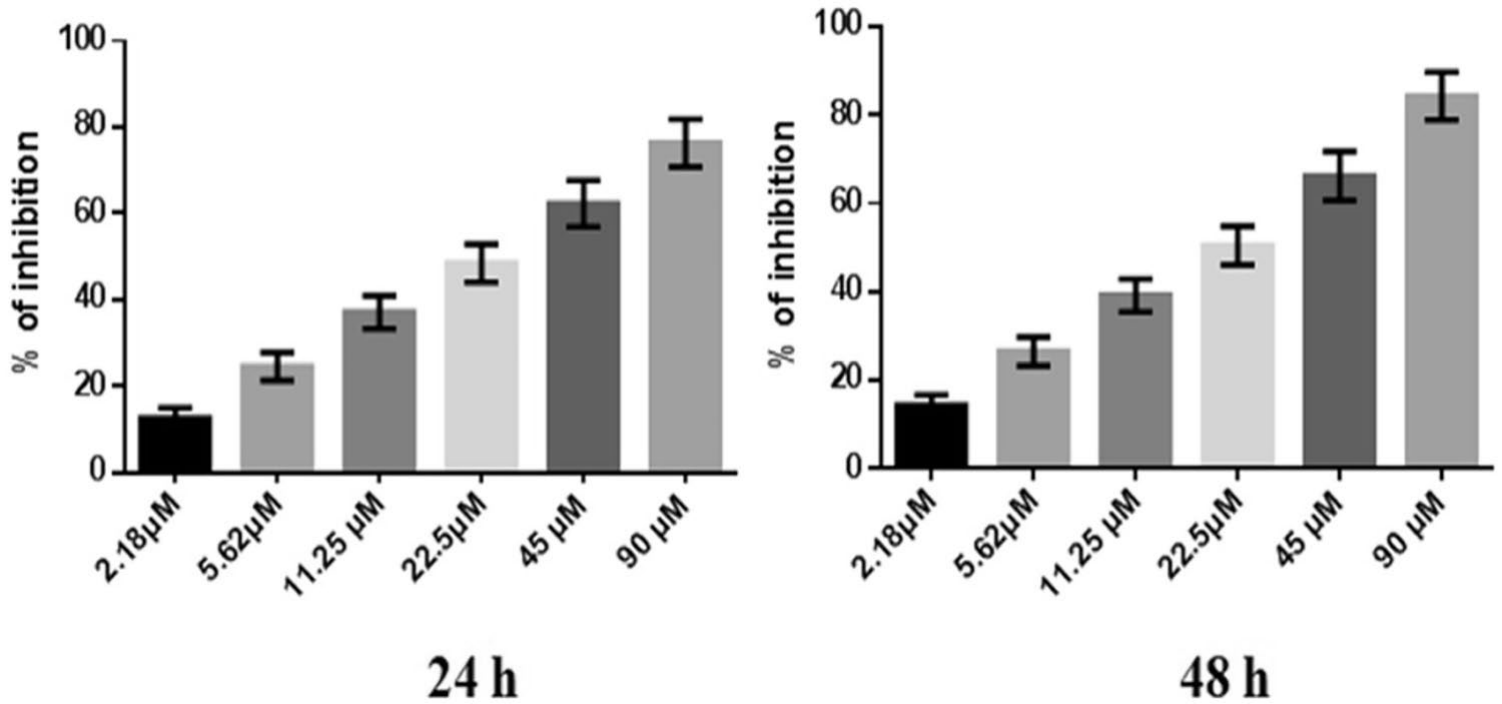
Anticancer efficacy of CYL on breast cancer cells at 24 h and 48 h. All values are displayed as the mean ± SEM of triplicate samples. The data were analyzed by one-way ANOVA followed by Tukey’s test

However, the percentage of inhibition of CYL at a maximum concentration of 90 µM showed 76.29 ± 5.52% and 84.29 ± 5.4% at 24 h and 48 h, respectively. The IC_50_ of CYL was found to be 3.9 µM at 24 h and 3.54 µM at 48 h (Fig. 4).

#### Anticancer efficacy of the combination treatment of NJ or DNG with CYL

**Fig. 5:**
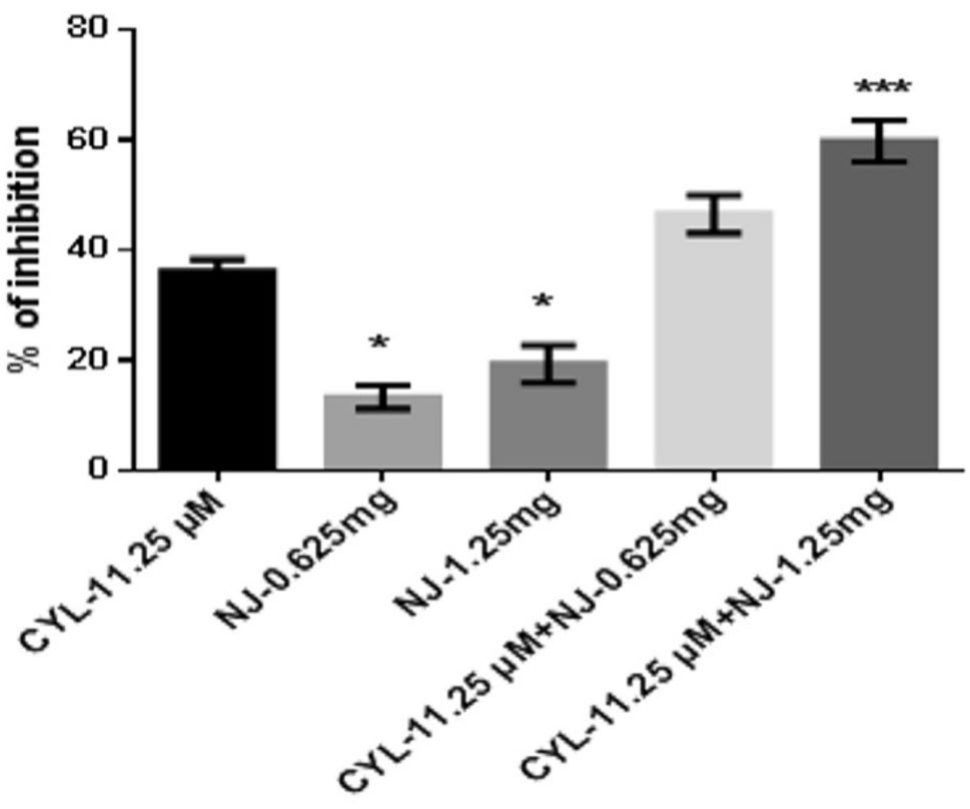
Anticancer efficacy of the combination treatment of NJ and CYL on breast cancer cells at 48 h. All values are displayed as the mean ± SEM of triplicate samples. The data were analyzed by one-way ANOVA followed by Tukey’s test; comparisons were performed with CYL-11.25; *p < 0.05, ***p < 0.01

At 48 h, the percentage of inhibition of NJ-0.625 mg and NJ-1.25 mg was found to be 13.2 ± 2.1% and 19.21 ± 3.4% respectively, whereas the percentage of inhibition of CYL-11.25 µM was found to be 36.08 ± 2.2%. Combination treatment of CYL-11.25 µM + NJ-0.625 mg did not find significant effect, while combination treatment of CYL-11.25 µM + NJ-1.25 mg significantly increased the percentage of inhibition to 59.8 ± 3.7% compared to treatment with CYL-11.25 µM (36.08 ± 2.2%) alone at 48 h (Fig. 5).

**Fig. 6:**
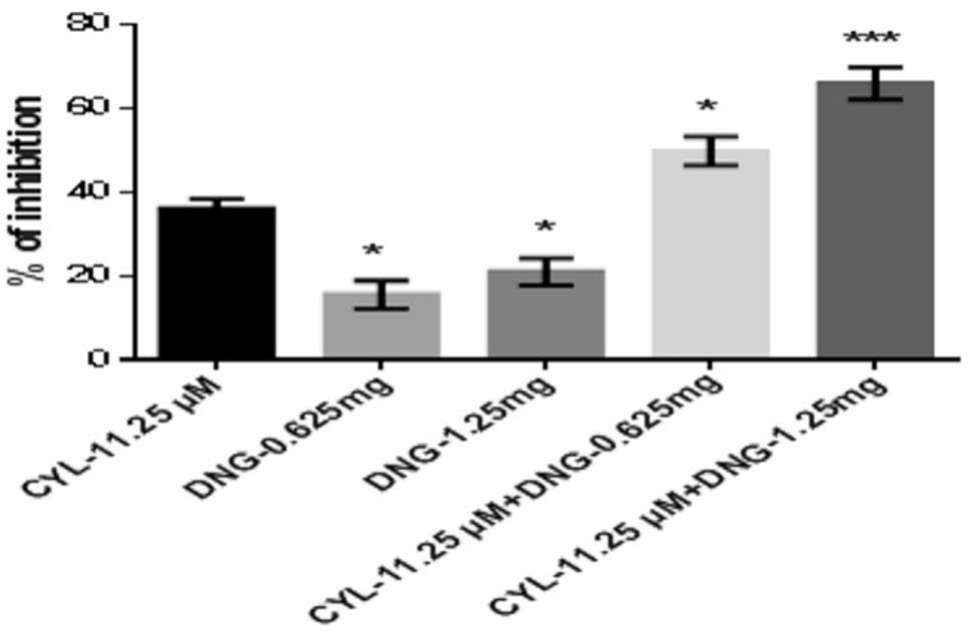
Anticancer efficacy of the combination treatment of DNG and CYL on breast cancer cells at 48 h. All the values are displayed as the mean ± SEM of triplicate samples. The data were analyzed by one-way ANOVA followed by Tukey’s test; comparisons were performed with CYL-11.25; *p < 0.05, ***p < 0.01

However, the percentage of inhibition of DNG-0.625 mg and DNG-1.25 mg was found to be 15.52 ± 3.3% and 21.0 ± 3.2%, respectively at 48 h, whereas the combination treatment of both CYL-11.25 µM + DNG-0.625 mg and CYL-11.25 µM + DNG-1.25 mg significantly increased the percentage of inhibition to 49.75 ± 3.4% and 65.88 ± 3.8%, respectively, compared to CYL-11.25 µM (36.08 ± 2.2%) alone treatment at 48 h (Fig. 6).

### Anticancer activity of NJ and DNG in tumor-bearing mice

#### Analysis of MST

**Fig. 7:**
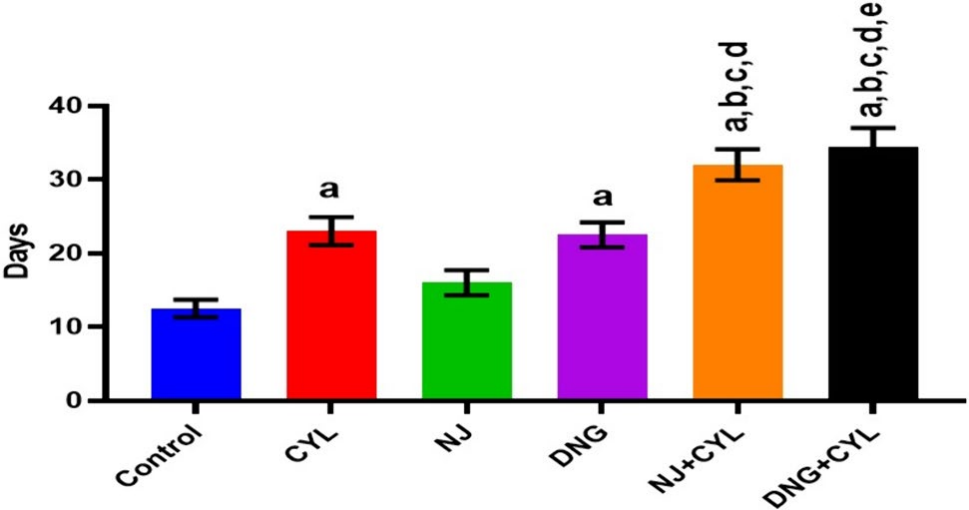
Effects of NJ and DNG on the MST following CYL treatment in tumor-bearing mice. All the values are displayed as the means ± SEMs of ten mice. The data were analyzed by one-way ANOVA followed by multiple test; ^a^*p* < 0.05 compared to the control; ^b^*p* < 0.05 compared to the CYL; ^c^*p* < 0.05 compared to the NJ; ^d^*p* < 0.05 compared to the DNG; and ^e^*p* < 0.05 compared to the NJ + CYL

The MST of the control EAC-bearing mice was 12.5 ± 1.2 days. However, compared with the control treatment, the CYL and DNG treatments significantly extended the MST to 23.0 ± 1.9 and 22.5 ± 1.7 days, respectively. Treatment with NJ alone did not significantly differ. The combination treatment of NJ + CYL and DNG + CYL significantly improved the MST to 32.0 ± 2.1 and 34.5 ± 2.5 days, respectively, compared to that of the CYL alone treatment group, while the combination DNG + CYL had more significant anticancer efficacy than did NJ + CYL (Fig. 7).

#### Percentage ILS analysis

**Fig. 8:**
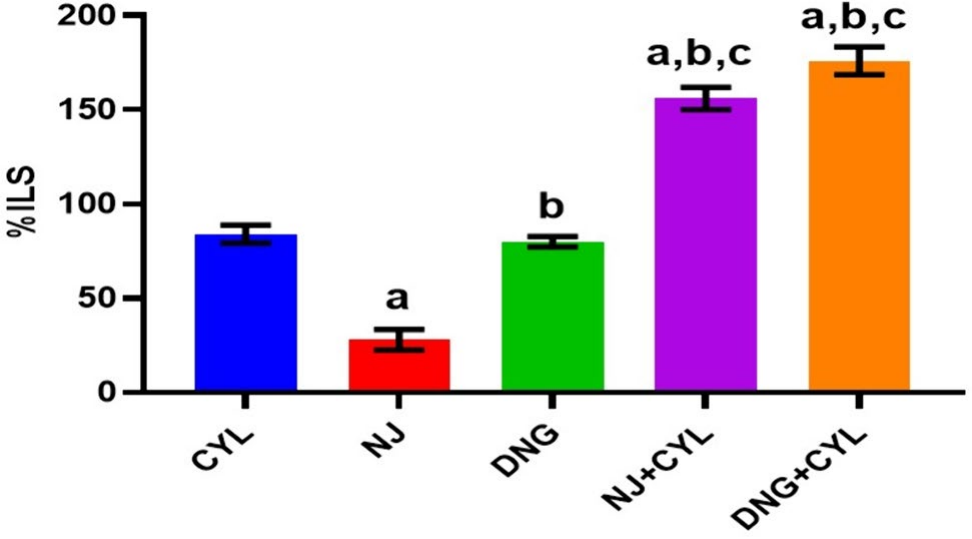
Effect of NJ and DNG on the %ILS following CYL treatment in tumor-bearing mice. All the values are expressed as the means ± SEMs of ten mice. The data were analyzed by one-way ANOVA followed by multiple test; ^a^*p* < 0.05 compared to CYL, ^b^*p* < 0.05 compared to NJ, ^c^*p* < 0.05 compared to DNG, and ^d^*p* < 0.05 compared to NJ + CYL

The %ILS in the CYL, NJ, and DNG alone treatment groups were 84.0 ± 4.7%, 28.0 ± 5.4%, and 80.0 ± 2.8%, respectively. However, compared with CYL alone, the combination of NJ + CYL and DNG + CYL significantly increased the %ILS to 156.0 ± 5.9% and 176.0 ± 7.4%, respectively (Fig. 8).

#### Survival analysis

**Fig. 9:**
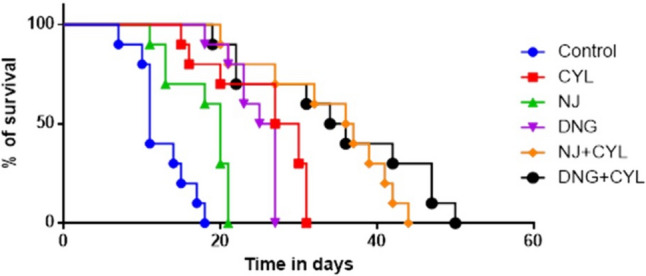
Effects of NJ and DNG on the Kaplan‒Meier estimate of survival in EAC-bearing mice following CYL treatment

The EAC mice in the control group survived up to 18 days. The EAC-bearing mice that were subjected to the CYL, NJ, and DNG treatments survived longer than did the control mice, which were up to 31, 21, and 27 days, respectively. NJ + CYL and DNG + CYL prolonged the survival rate by up to 44 and 50 days, respectively (Fig. 9).

#### Viable cell count analysis

**Fig. 10:**
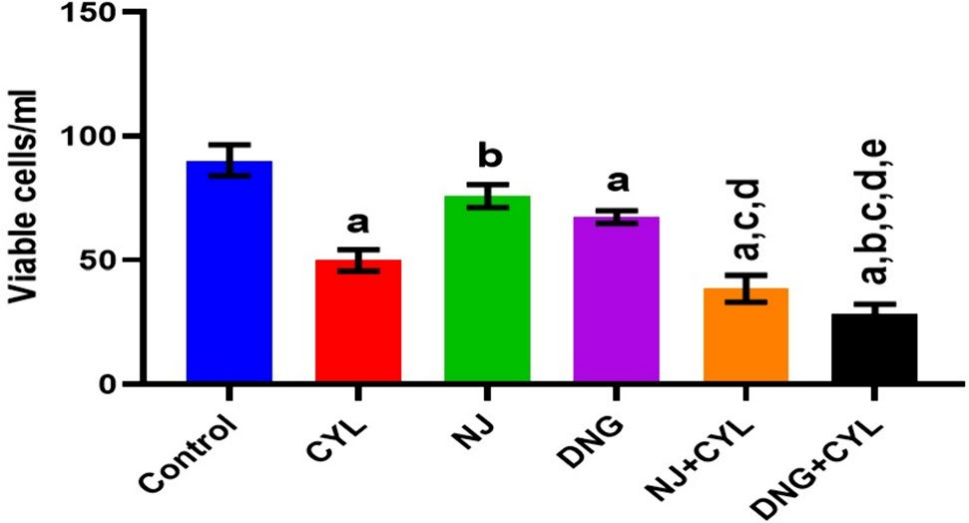
Effect of NJ and DNG on the viable cell count following CYL treatment in tumor-bearing mice. All the values are expressed as the means ± SEMs of ten mice. The data were analyzed by one-way followed by Tukey’s multiple test; ^a^*p* < 0.01 compared to the control, ^b^*p* < 0.01 compared to CYL, ^c^*p* < 0.01 compared to NJ, ^d^*p* < 0.01 compared to DNG, and ^e^*p* < 0.01 compared to NJ + CYL

The percentage of viable cells in the control group was 90.2 ± 6.3/ml. Treatment with CYL, NJ, or DNG significantly decreased the viable cell count to 49.98 ± 4.24/ml, 75.78 ± 4.65/ml, and 67.35 ± 2.54/ml, respectively, compared to that in the control group. However, the combination of NJ + CYL and DNG + CYL significantly decreased the viable cell count to 38.65 ± 5.45/ml and 28.25 ± 4.21/ml, respectively, compared to that in the CYL-treated group, whereas the DNG + CYL group showed greater viability than that of the NJ + CYL group (Fig. 10).

#### Non-viable cell count analysis

**Fig. 11:**
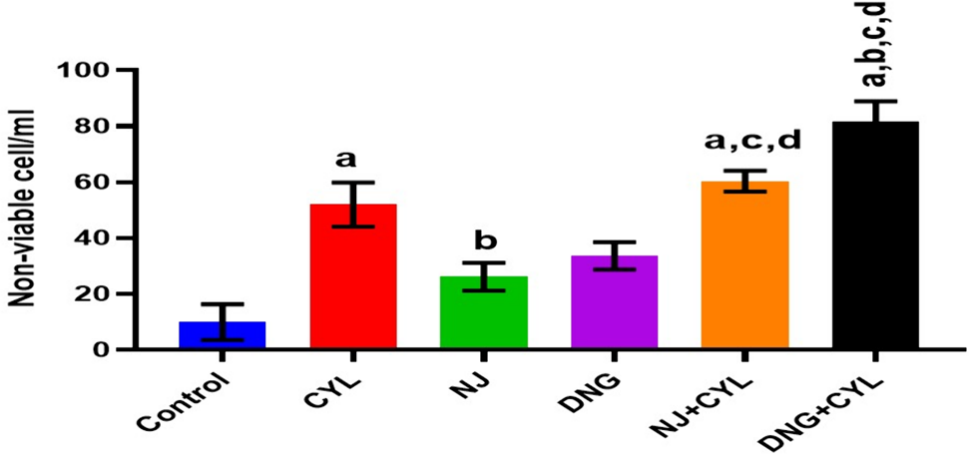
Effects of NJ and DNG on the non-viable cell count following CYL treatment in tumor-bearing mice. All the values are expressed as the means ± SEMs of ten mice. The data were analyzed by one-way ANOVA followed by Tukey’s multiple comparisons test; ^a^*p* < 0.01 compared with the control, ^b^*p* < 0.01 compared with the CYL treatment, ^c^*p* < 0.01 compared with the NJ treatment, ^d^*p* < 0.01 compared with the DNG treatment, and ^e^*p* < 0.01 compared with the NJ + CYL treatment

The percentage of non-viable cells in the control group was 9.99 ± 6.36/ml. Treatment with CYL, NJ, or DNG significantly increased the non-viable cell count to 52.02 ± 7.89/ml, 26.22 ± 5.02/ml, and 33.65 ± 4.89/ml, respectively, compared to that of the control. However, compared with the CYL alone, the combination of NJ + CYL and DNG + CYL significantly improved the non-viable cell count to 60.35 ± 3.75/ml and 81.75 ± 7.21/ml, respectively (Fig. 11).

#### Tumor volume analysis

**Fig. 12:**
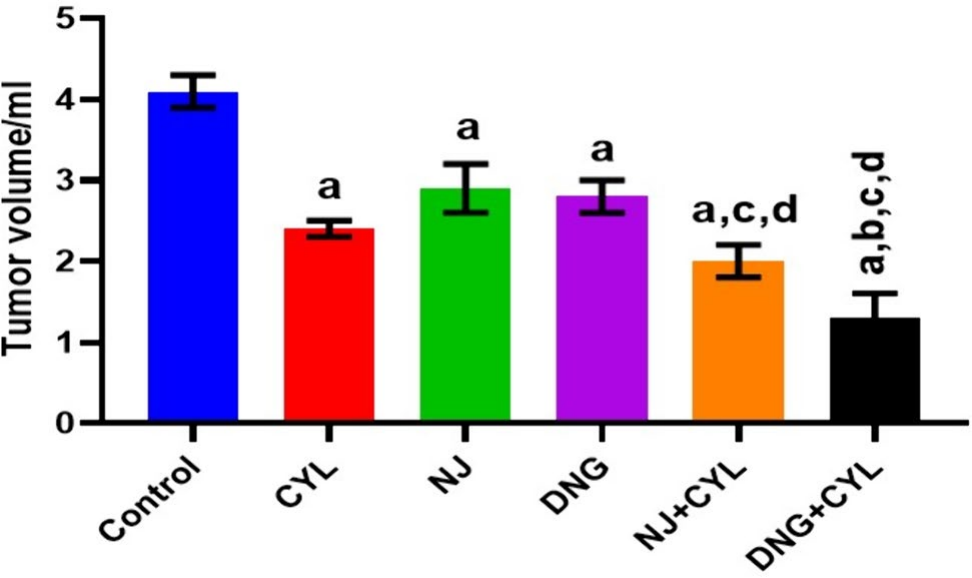
Effect of NJ and DNG on tumor volume following CYL treatment in tumor-bearing mice. All the values are expressed as the means ± SEMs of ten mice. The data were analyzed by one-way ANOVA followed by multiple test; ^a^*p* < 0.05 compared to the control, ^b^*p* < 0.05 compared to the CYL treatment, ^c^*p* < 0.05 compared to the NJ treatment, ^d^*p* < 0.05 compared to the DNG treatment, and ^e^*p* < 0.05 compared to the NJ + CYL treatment

In the control group, the tumor volume was 4.1 ± 0.2 ml. The tumor volume significantly decreased to 2.4 ± 0.1 ml, 2.9 ± 0.3 ml, and 2.8 ± 0.2 ml in the CYL, NJ, and DNG treatment groups, respectively, compared to that in the control group. In the DNG + CYL combination treatment group, the tumor volume significantly decreased to 1.3 ± 0.3 ml in comparison to that in the CYL-treated group. However, compared with the CYL treatment alone, the combination treatment of NJ + CYL did not significantly differ (Fig. 12).

### Body weight analysis

**Fig. 13:**
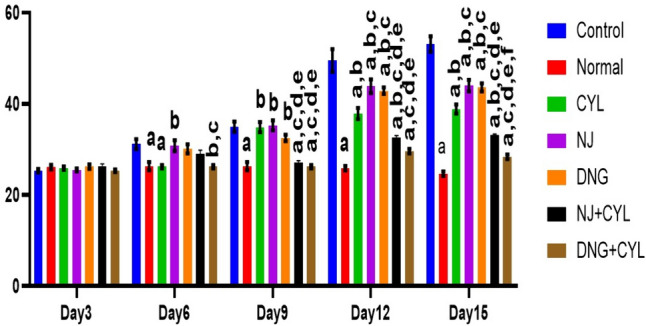
Effects of NJ and DNG on body weight following CYL treatment in tumor-bearing mice. All the values are displayed as the means ± SEMs, *n* = 10. Data were analyzed by two-way ANOVA' followed by Tukey's multiple' comparisons' test; ^a^*p* < 0.01 compared to control, ^b^*p* < 0.01 compared to normal, ^c^*p* < 0.01 compared to CYL, ^d^*p* < 0.01 compared to NJ, ^e^*p* < 0.01 compared to DNG, ^f^*p* < 0.01 compared to NJ + CYL

Analysis of body weight was performed every 3 days for 15 days. In the control group, body weight significantly increased from 6 to 15 days. On day 15, the body weight of the control group significantly increased to 53.1 ± 1.7 g compared to that of the normal group (24.7 ± 0.5 g). However, compared with the control treatment, the individual treatments with CYL, NJ, and DNG significantly decreased the body weight on days 12 and 15. However, the combination treatment of NJ + CYL and DNG + CYL markedly decreased the body weight compared to that of the CYL-treated group. However, DNG + CYL significantly decreased body weight compared to that of NJ + CYL on day 15 (Fig. 13).

### Hematological parameter analysis

Compared with those in the normal group, the RBC and Hb levels and the WBC count in the EAC control group were significantly lower. Similarly, compared with the control treatment, the individual treatment with CYL markedly decreased RBC, WBC, and Hb levels. Individual treatment with NJ, as well as DNG, significantly increased the RBC count and Hb content compared to those in the control and CYL alone treatment groups. Similarly, individual treatment with NJ or DNG significantly decreased the WBC count compared to that in the control group. Compared with the CYL treatment, the combination of NJ + CYL and DNG + CYL significantly improved the RBC and Hb levels and decreased the WBC count. However, compared with NJ + CYL, DNG + CYL significantly increased RBC and Hb levels. All the data are presented in Table 3.

**Table 3:** Effects of NJ and DNG on hematological parameters following CYL treatment in tumor-bearing mice

Treatment groups	RBC (million/mm^3^)	WBC (cells/mm^3^)	Hb (g/dl)
Normal	12.40 ± 0.3	11,200.0 ± 350.1	11.6 ± 0.5
Control	7.50 ± 0.2^a^	17,750.0 ± 477.1^a^	7.1 ± 0.2^a^
CYL	2.00 ± 0.2^a,b^	14,524.2 ± 510.2^a,b^	3.1 ± 0.4^a,b^
NJ	8.90 ± 0.3^a,b,c^	15,800.1 ± 322.0^a,b^	9.6 ± 0.2^a,b,c^
DNG	8.93 ± 0.4^a,b,c^	15,600.0 ± 407.1^a,b^	9.8 ± 0.2^a,b,c^
NJ + CYL	6.20 ± 0.2^a,c,d,e^	12,440.0 ± 349.2^b,c,d,e^	6.0 ± 0.3^a,c,d,e^
DNG + CYL	9.40 ± 0.3^a,b,c,f^	12,120.2 ± 502.1^b,c,d,e^	7.80 ± 0.5^a,c,d,e,f^

All the values are expressed as the means ± SEMs of six mice. Data were analyzed through one-way ANOVA followed by Tukey’s' multiple comparisons' test

^a^*p* < 0.01 compared to normal

^b^*p* < 0.01 compared to control

^c^*p* < 0.01 compared to CYL

^d^*p* < 0.01 compared to NJ

^e^*p* < 0.01 compared to DNG

^f^*p* < 0.01 compared to NJ + CYL

#### Serum biochemical marker analysis

As shown in Table 4, the serum biochemical parameters, i.e., ALT, AST, and ALP, were significantly greater in the control group than those in the normal group. Treatment with CYL did not significantly change the ALT, AST, or ALP levels compared to those in the control group. Individual treatment with NJ did not significantly change the serum ALT, AST, or ALP levels compared to those in the control group, but DNG significantly decreased the serum ALT, AST, and ALP levels. However, compared with CYL treatment, combination therapy with NJ + CYL and DNG + CYL significantly decreased alanine transaminase (ALT), aspartate transaminase (AST), and alkaline phosphatase (ALP) levels. The overall data are presented in Table 4.

**Table 4:** Effects of NJ and DNG on the serum biochemical parameters of tumor-bearing mice following CYL treatment

Treatment groups	ALT (U/L)	AST (U/L)	ALP (U/L)
Normal	58.70 ± 2.6	50.12 ± 2.1	40.20 ± 3.5
Control	79.65 ± 1.9^a^	80.23 ± 0.9^a^	70.32 ± 1.5^a^
CYL	81.55 ± 1.5^a,b^	79.65 ± 0.9^a,b^	70.33 ± 3.1^a,b^
NJ	75.42 ± 0.9^a^	80.01 ± 2.8^a,c^	69.40 ± 2.2^a,c^
DNG	74.00 ± 1.3^a,b,c^	72.0 ± 3.9^a,b,c^	65.23 ± 1.5^a,b,c^
NJ + CYL	58.99 ± 1.2^b,c,d,e^	59.36 ± 2.4^b,c,d^	56.50 ± 2.1^a,b,d,e^
DNG + CYL	58.07 ± 0.7^b,c,d,e^	55.64 ± 2.1^b,c,d^	46.75 ± 1.2^a,b,c,d,e^

All the values are displayed as the means ± SEMs of six mice. Data were analyzed by one-way ANOVA followed by Tukey’s' multiple comparisons' test

^a^*p* < 0.01 compared to normal

^b^*p* < 0.01 compared to control

^c^*p* < 0.01 compared to CYL

^d^*p* < 0.01 compared to NJ

^e^*p* < 0.01 compared to DNG

^f^*p* < 0.01 compared to NJ + CYL

### Histopathological analysis of liver tissue

**Fig. 14:**
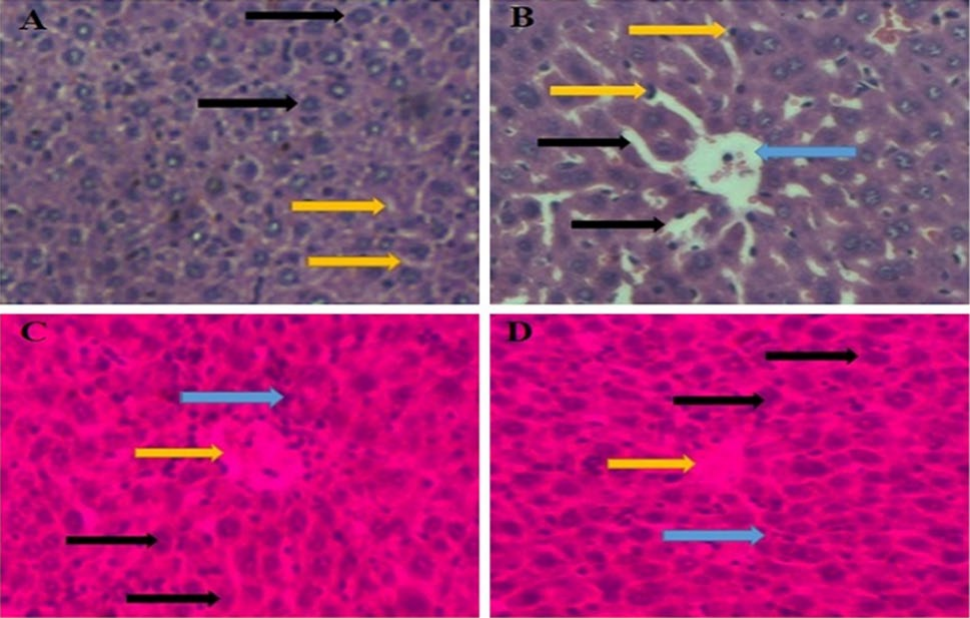
**A**. A vehicle-treated mouse showing a healthy liver section. A liver section from a mouse showing the usual sinusoidal space (yellow arrow) and healthy hepatocytes with a polygonal shape (black arrow) [hematoxylin- and eosin-stained section H&E X 400]. **B**. In the CYL (40 mg/kg) group, a liver section from a mouse exhibiting sinusoidal dilatation (black arrow), periportal inflammation, and an asymmetrical portal vein (blue arrow), lymphocytes between hepatocytes (yellow arrow), and small necrotic hepatocytes (blue arrow) (hematoxylin- and eosin-stained section H&E X 400). **C**. The combination treatment of NJ + CYL had protective effects and minimized toxicity. The liver section of a mouse pre-treated with 360 mg/kg NJ along with CYL displaying healthy hepatocytes (black arrows) and portal space with mild-to-moderate inflammation (yellow arrow) plus mild necrotic hepatocytes (blue arrow) [hematoxylin- and eosin-stained section H&E X 400]. **D**. The combination treatment of DNG + CYL had protective effects and minimized toxicity. A liver section of a mouse pre-treated with 360 mg/kg DNG and CYL showing healthy hepatocytes (black arrow), healthy portal vein tissue with mild-to-moderate inflammation (yellow arrow), and mild necrotic minor hepatocytes (blue arrow) (hematoxylin- and eosin-stained section H&E X 400)

Histopathological examination of mouse livers was performed under a light microscope after consecutive 14 days of treatment with CYL (40 mg/kg) or noni (360 mg/kg), and the results are shown in Fig. 14A–D. Vehicle-treated mice were used to establish healthy liver sections. Sections of liver tissue from healthy sinusoidal spaces and healthy hepatocytes were obtained (Fig. 14A). In the CYL (40 mg/kg) group, liver sections from the mice exhibited sinusoidal dilatation, periportal inflammation, an asymmetrical portal vein, and lymphocytes between hepatocytes, as well as small necrotic hepatocytes (Fig. 14B). The combination treatment of NJ + CYL had protective effects and minimized toxicity. Liver sections from a mouse pre-treated with a combination of NJ and CYL showed healthy hepatocytes and portal space with mild-to-moderate inflammation and mild necrotic minor hepatocytes (Fig. 14C). The combination of DNG + CYL had protective effects and minimized toxicity. Liver sections from an animal pre-treated with 360 mg/kg DNG and CYL showed healthy hepatocytes; healthy portal veins, including mild-to-moderate inflammation; and mild necrotic small hepatocytes (Fig. 14D).

## Discussion

Cyclophosphamide is an alkylating anticancer drug that has immune-suppressive effects (Moore 1991). Cyclophosphamide is effective in treating a broad spectrum of malignancies, i.e., leukemia, lymphoma, and prostate, breast, lung, and ovarian cancers (Khan et al. 2004; Cui et al. 2016). A high dose of CYL is known to induce severe oxidative stress in healthy tissues, which can lead to various complications in cancer patients (Pushpangadan and Subramonian 1998). The current study was designed to assess the anticancer activity of CYL in the presence of noni in transplantable tumor-bearing mice. A large number of herbal plants are used in the Indian traditional system of medicine to treat cancer; however, many of them still have not been scientifically proven (Sharma et al. 2016). Hence, identifying potent anticancer plants is important. Earlier studies reported that *Morinda citrifolia* fruit extract (MCF-7) showed potent anticancer activity in breast cancer cell lines (Dewys 1982).

In the present in vitro and in vivo* studies*, we investigated the ability of noni (NJ and DNG) to enhance the anticancer activity of CYL in cell culture. We observed that NJ, DNG, and CYL inhibited the growth of breast cancer cells (MDA-MB-468) in a dose- and time-dependent manner at 24 h and 48 h. The percentage of growth inhibition in response to DNG was greater than that in response to NJ. The IC_50_ values of NJ were found to be 9.17 mg/ml and 8.75 mg/ml (Fig. 2); the IC_50_ values of DNG were found to be 6.33 mg/ml and 5.44 mg/ml (Fig. 3); and the IC_50_ values of CYL were found to be 3.9 mg/ml and 3.54 mg/ml (Fig. 4) at 24 and 48 h, respectively. The obtained IC_50_ values indicate that both NJ and DNG have potent anticancer activity and that DNG is more potent than NJ. In addition, NJ and DNG in combination with CYL had greater inhibitory effects on tumor growth than those of CYL alone. These findings suggested that the ability of CYL to inhibit tumor growth could be strengthened by noni supplementation. With respect to combination therapy, we found that, compared with CYL-11.25 µM alone, CYL-11.25 µM + NJ-1.25 mg significantly improved the percentage of inhibition at 48 h (Fig. 5). However, both the combination of CYL-11.25 µM + DNG-0.625 mg and CYL-11.25 µM + DNG-1.25 mg significantly improved the percentage of inhibition compared to CYL-11.25 µM alone at 48 h (Fig. 6), indicating that noni can enhance the antitumor activity of CYL when given in combination therapy. These outcomes may be due to the polysaccharide and phytosterol contents being generated through immune-stimulating activity. Our in vitro findings are consistent with those of Dewy (1982), who stated that phytosterol compounds can enter the cell membrane, leading to changes in membrane fluidity and alterations in the functions of membrane-bound enzymes. They change signal transduction pathways, which leads to the induction of tumor cell apoptosis. Moreover, these compounds can improve peripheral blood lymphocyte counts in humans and T cell proliferation, as shown in an in vitro study that indicated a potential increase in immune system strength (Meysam et al. 2017). Our in vitro* findings* also correlate with the fact that curcumin alone and in combination significantly augment the cytotoxicity of paclitaxel, vincristine, and methotrexate in GIT cancer-infected cells (Ueno et al. 2006).

A fruitful anticancer drug should kill cancer cells without inducing injury to healthy cells. Certain substances in herbs are well known to kill cancer cells but not healthy cells. Therefore, it is essential to screen and isolate apoptotic inducers from herbs, either in the form of crude extracts or as pharmacologically active compounds. Nevertheless, natural phytoconstituents act as feasible sources of drugs or supplements worldwide (Manda and Bhatia 2003). Cyclophosphamide causes oxidative stress through the generation of free radicals in tissues and cells (Hirazumi and Furusawa 1999). The above in vitro findings corroborate the results of the present in vivo study. The present animal study revealed that, compared with the control treatment, NJ and DNG treatment significantly augmented the life span and survival rate of cancer mice (Figs. 8, 9). Coadministration of NJ or DNG with CYL hindered cell division, which indicated a decrease in cell viability (Fig. 10), prompted augmentation of non-viable cells (Fig. 11), and increased tumor volume (Fig. 12), which contributed to the increased survival time of the mice (Fig. 6) and suggested the antiproliferative effects of NJ and DNG.

Body weight is one of the most essential parameters for assessing tumor growth. An increase in body weight was found in the control group. Body weight was significantly decreased in CYL, NJ, and DNG alone-treated groups when compared to the control group, while combination showed better results compared to CYL alone (Fig. 13). Our findings are consistent with those of Hirazumi and Furusawa (1999), who suggested that noni juice improved life span and suppressed tumor growth when combined with doxorubicin, cisplatin, 5-fluorouracil, or vincristine through activation of the hostׄ immune system, suggesting that noni juice is a promisingׄ supplemental agentׄ for cancer treatment (Jones and AbuMweis 2009).

Numerous medicinal plants and herbs containing various phytoconstituents, including phenolic and flavonoid compounds, carotenoids and alkaloids, confer defensive effects against various cancers. Noni fruits are a rich source of these components and have been demonstrated to have a wide variety of health benefits; in addition, they are used for the management of various ailments, including cancer. Noni kills cancer cells by several mechanisms, such as inhibiting angiogenesis, preventing carcinogen-DNA adduct formation or activating immune responses, exerting antioxidant effects, preventing natural killer cells from multiplying into malignant tumors, and preventing the formation of oncogenes. Flavonoids have a chemopreventive effect on cancer through their ability to transduce signals that affect cell proliferation and angiogenesis (Hohl 1996). The cytotoxicity of these compounds in healthy cells, as well as their anticancer properties, may be due to the presence of flavonoids. Noni juice contains limonene, a phytoconstituent that stimulates the thymus gland to release a greater number of T cells that destroy cancer cells (He et al. 2021). A previous study reported that myelosuppression and anemia are common complications of cancer chemotherapy because oxidative stress in the bone marrow leads to deterioration of the hematopoietic system (Hasenclever and Diehl 1998). Elevated WBC counts and abridged RBC counts and Hb levels were also found in the EAC control group in the present study; these changes were reversed by NJ and DNG in combination with CYL, and the Hb levels were maintained close to normal, indicating that noni has hematopoietic protective effect (Table 3). The enhancement in the hematological profile of tumor-bearing mice following treatment with NJ and DNG could be due to the phenolic and flavonoid compounds having antioxidant activity. Ascetic carcinoma induces iron deficiency anemia under various conditions, such as hemolytic or myelopathic conditions (Morton and George 2016).

A decrease in the concentration of Hb is a common disorder in cancer patients who triggers abnormal physical function, leading to decreased quality of life, including fatigue and cognitive deficits (Jiang et al. 2007). Current outcomes overlap with the detail that cancer has been known for several centuries as a cause of anemia and thrombocytopenia (Coussens and Werb 2002). An earlier study reported that developing tumor encumbrance is associated with deterioration of the immune system. The WBCs of the inborn body immune system, comprising neutrophils, macrophages, and NK cells, penetrate the tumor to induce a multifaceted killing response (DiCarlo et al. 2001). There was a statistically significant increase in the number of WBCs as well as neutrophils in tumor-bearing animals because these blood cells are the first cells to reach the tumor where they release chemokines and proteases, which can induce immune effector cells (Bodansky and Schwartz 1962). Blood serum enzymes have been documented for many years as probable initial indicators of cancer growth and help in the development and deterioration of disease (Thapa and Walia 2007). Liver function tests have been commonly used to detect liver damage. The serum ALT, ALP, and AST levels are the most common hepatocellular markers used to analyze hepatocellular injury (Mondal et al. 2013).

Hepatotoxicity may occur owing to a cytotoxic agent or/or possibly its noxious substances. Significant elevations in the levels of alanine transaminase (ALT) and aspartate transaminase (AST), including proteins, reveal the hepatocellular injuries caused by several substances. Serum biochemical data indicated that hepatotoxicity developed during EAC inoculation. It has been stated that the development of tumors in humans, as well as in experimental animals, is well known to interrupt numerous essential organs, particularly the liver (Sallie et al. 1991). These findings are in agreement with those of Tangpong et al. (2011), who reported that *Garcinia cambogia*, which contains flavonoid compounds, has anticancer effects and can prevent doxorubicin-induced neurotoxicity. In addition, a recent study reported the use of the fruit extract of *Garcinia cambogia* (Shivapriya et al. 2013), which consists of phytoconstituents such as garcinol and has good antioxidant and anticarcinogenic activity. In the present study, biochemical measurements of alanine transaminase (ALT), aspartate transaminase (AST), and alkaline phosphatase (ALP) levels revealed that some degree of hepatotoxicity was related to the inoculation of EAC cells. Treatment with NJ and DNG restored the elevated biochemical markers within the normal range, while combination treatment with CYL was associated with better outcomes than treatment with CYL alone (Table 4), indicating that noni protects the liver against EAC-induced hepatotoxicity. Histopathological analysis of mouse livers confirmed that CYL induced hepatotoxicity in mice (Fig. 14B), which could be protected or minimized by nasal supplement therapy in combination with CYL in EAC tumor-bearing mice (Fig. 14C, D). The greater potency of DNG than that of NJ may be due to the presence of *Garcinia cambogia* fruit extract, which is likely due to the presence of additional phytochemical constituents with antioxidant activity.

## Conclusions

In conclusion, the combination of CYL with noni has a better antitumor effect than that of either noni or CYL alone in both in vitro and in vivo studies. Moreover, noni can prevent the disruption of hematological parameters and serum biochemical marker levels induced by EAC cells and CYL. Therefore, these findings demonstrated that noni can minimize myelotoxicity and hepatotoxicity induced by EAC and CYL cells in tumor-bearing mice. Overall, these studies demonstrated that DNG had a greater effect than NJ. Therefore, this study suggests that DNG is a better candidate for use as a supplemental therapy to enhance the anticancer effect of CYL and to reduce the myelosuppression and hepatic damage side effects caused by EAC cells and CYL in tumor-bearing mice. Further mechanistic and molecular studies are in progress.

## Data Availability

All the data generated or analyzed during this study are included in this article.

## Abbreviations

CYL: Cyclophosphamide
NJ: Noni juice
DNG: Divine noni gold
SRB: Sulforhodamine-B
EAC: Ehrlich ascites carcinoma
MST: Mean survival time
ILS: Increase in life span
RBC: Red blood cell
WBC: White blood cell
AST: Aspartate transaminase
ALT: Alanine transaminase
ALP: Alkaline phosphatase

## Declarations

### ARRIVE guidelines

The study was approved by the Institutional Animal Ethical Committee (IAEC No. 162/2016) of JSS College of Pharmacy and was reported in accordance with ARRIVE guidelines.

### “IUCN policy statement on research involving species at risk of extinction” statement

The authors hereby declare that they comply with the IUCN Policy Statement on Research Involving Species at Risk of Extinction and the Convention on the Trade in Endangered Species of Wild Fauna and Flora.
